# EFFECT OF RADIATION DOSE LEVEL ON ACCURACY AND PRECISION OF MANUAL SIZE MEASUREMENTS IN CHEST TOMOSYNTHESIS EVALUATED USING SIMULATED PULMONARY NODULES

**DOI:** 10.1093/rpd/ncw041

**Published:** 2016-06-07

**Authors:** Christina Söderman, Åse Allansdotter Johnsson, Jenny Vikgren, Rauni Rossi Norrlund, David Molnar, Angelica Svalkvist, Lars Gunnar Månsson, Magnus Båth

**Affiliations:** 1 Department of Radiation Physics, Institute of Clinical Sciences, The Sahlgrenska Academy at University of Gothenburg, SE-413 45 Gothenburg, Sweden; 2 Department of Radiology, Institute of Clinical Sciences, The Sahlgrenska Academy at University of Gothenburg, SE-413 45 Gothenburg, Sweden; 3 Department of Radiology, Sahlgrenska University Hospital, SE-413 45 Gothenburg, Sweden; 4 Department of Medical Physics and Biomedical Engineering, Sahlgrenska University Hospital, SE-413 45 Gothenburg, Sweden

## Abstract

The aim of the present study was to investigate the dependency of the accuracy and precision of nodule diameter measurements on the radiation dose level in chest tomosynthesis. Artificial ellipsoid-shaped nodules with known dimensions were inserted in clinical chest tomosynthesis images. Noise was added to the images in order to simulate radiation dose levels corresponding to effective doses for a standard-sized patient of 0.06 and 0.04 mSv. These levels were compared with the original dose level, corresponding to an effective dose of 0.12 mSv for a standard-sized patient. Four thoracic radiologists measured the longest diameter of the nodules. The study was restricted to nodules located in high-dose areas of the tomosynthesis projection radiographs. A significant decrease of the measurement accuracy and intraobserver variability was seen for the lowest dose level for a subset of the observers. No significant effect of dose level on the interobserver variability was found. The number of non-measurable small nodules (≤5 mm) was higher for the two lowest dose levels compared with the original dose level. In conclusion, for pulmonary nodules at positions in the lung corresponding to locations in high-dose areas of the projection radiographs, using a radiation dose level resulting in an effective dose of 0.06 mSv to a standard-sized patient may be possible in chest tomosynthesis without affecting the accuracy and precision of nodule diameter measurements to any large extent. However, an increasing number of non-measurable small nodules (≤5 mm) with decreasing radiation dose may raise some concerns regarding an applied general dose reduction for chest tomosynthesis examinations in the clinical praxis.

## INTRODUCTION

Chest tomosynthesis refers to the technique of acquiring a number of projection radiographs within a limited angular interval around a patient and using these radiographs to reconstruct section images of the chest containing much less of the overlying anatomy present in the original radiographs^(1–4)^. Compared with conventional radiography, tomosynthesis has been shown to increase the sensitivity for different types of subtle lung lesions^(5–11)^. Benefits when compared with computed tomography (CT) include lower patient radiation dose^(12, 13)^ and better patient throughput^(14, 15)^. This makes it an interesting potential alternative to CT for certain clinical tasks. One such task, which has been suggested previously by Dobbins and McAdams^(3)^, is the follow-up of incidentally detected pulmonary nodules, in which the size of the nodules is monitored in order to detect any growth suggesting malignancy^(16)^.

Previous findings have supported pulmonary nodule follow-up as a potential area of use for chest tomosynthesis. For example, in a study by Vikgren *et al.*^(7)^, all pulmonary nodules larger than 6 mm detected on CT images (5 mm slice thickness) included in the study were also visible in retrospect with chest tomosynthesis. Furthermore, previous studies have shown high accuracy of nodule size measurement with chest tomosynthesis as well as good agreement between nodule size measurements made in CT and chest tomosynthesis images^(17–20)^. Johnsson *et al.*^(17)^ performed a phantom study in which measurements were made on spheres in a non-anatomical background. The sizes of the spheres were known, making it possible to investigate possible systematic errors in the measurements. However, the non-anatomical background restricted the clinical validity of the results. Söderman *et al.*^(19)^ addressed this issue by placing artificial ellipsoid-shaped nodules with known sizes in clinical chest tomosynthesis images. A similar approach was used in a study by Shim *et al.*^(20)^, in which model nodules with known sizes were placed in an anthropomorphic chest phantom. In another study by Johnsson *et al.*^(18)^, measurements were made on real clinical nodules found in patients. The true sizes of the nodules were, however, not known. Limitations with chest tomosynthesis, in terms of low nodule visibility and decreased nodule size measurement accuracy, was identified by Söderman *et al.*^(19)^ for nodules at positions in the lung corresponding to locations in low dose areas of the acquired tomosynthesis projection radiographs. This was particularly evident for nodules located behind the heart or the diaphragm.

Regarding the dose level used for chest CT examinations, previously reported effective doses have ranged from 4 to 18 mSv^(7, 21)^. However, low-dose CT imaging protocols, resulting in a dose of 1–2 mSv^(22, 23)^, and even ultra-low-dose protocols, achievable using modern iterative reconstruction techniques and resulting in effective doses below 0.5 mSv^(24)^, have also been presented to be suitable for chest CT examinations. Reported levels of effective dose from a chest tomosynthesis examination have been in the range from 0.1 to 0.2 mSv^(8, 10, 12, 13)^. However, recent studies have shown that a reduction to an effective dose of 0.04 mSv for a chest tomosynthesis examination could be possible with remained levels of detection of pulmonary nodules^(25, 26)^. These findings indicate that a reduction in current, commonly used, dose levels in chest tomosynthesis may be considered for implementation in clinical praxis. If, at the same time, chest tomosynthesis is to be considered for use in pulmonary nodule follow-up, it is important to investigate the effect of a reduced radiation dose on nodule size assessment. Therefore, the aim of the present study was to investigate the dependency of the accuracy and precision of nodule diameter measurements on the radiation dose level in chest tomosynthesis. The study was performed using artificial nodules with known sizes placed in patient chest tomosynthesis images and simulating a dose reduction of the images. The study was restricted to nodules at positions in the lung corresponding to locations in high-dose areas of the tomosynthesis projection radiographs.

## MATERIALS AND METHODS

### Acquisition of patient images

The chest tomosynthesis images used in the present study were from patients referred for a combined chest radiography and chest CT examination. For study purposes, a chest tomosynthesis examination was included for these patients after approval by the Regional Ethical Review Board. All patients gave written informed consent.

The chest tomosynthesis examinations were performed with GE Definium 8000 with VolumeRAD option (GE Healthcare, Chalfont St Giles, UK). With this system, the detector is at a fixed position during the acquisition of the tomosynthesis radiographs, while the X-ray tube performs a continuous vertical movement within an angular interval of ±15° relative to the standard posteroanterior (PA) projection. Sixty projection radiographs are acquired at a tube voltage of 120 kVp. These settings have been shown to result in adequate image quality in the final reconstructed section images^(27)^. The tube load for each projection image is determined by multiplying the tube load used for a conventional PA radiograph scout image, acquired with automatic exposure control, with a user-adjustable dose ratio, and distributing the resulting tube load evenly over the 60 projection radiographs after rounding down to the closest Renard step. A restriction of a minimum tube load of 0.25 mAs per projection radiograph is applied. In the present study, a dose ratio of 10:1 was used. Using the 60 projection radiographs, the final section images of the chest are reconstructed in the coronal plane by filtered back-projection. For a standard-sized patient (170 cm/70 kg), the effective dose of a chest tomosynthesis examination, excluding the scout image, on this system has been reported to be 0.12 mSv^(13)^.

### Creation of artificial nodules

**Figure 1.**
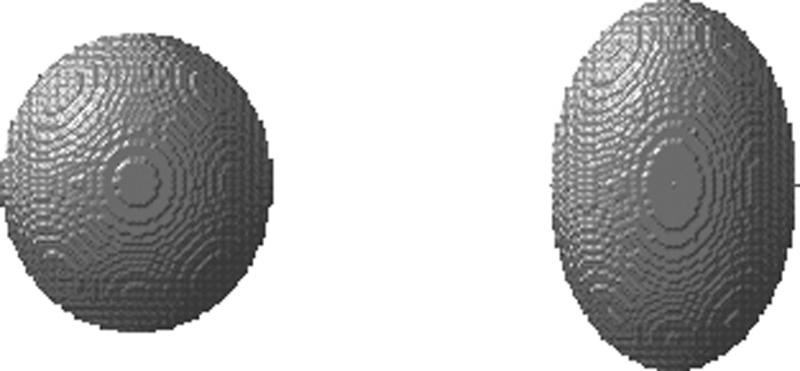
Examples of the different nodule shapes included in the study. For the nodule to the left, the ratio of the lengths of the major ellipsoid axis and minor ellipsoid axes is 1.1 and for the nodule to the right the same ratio is 1.5.

In total, 81 artificial three-dimensional nodules were generated. All nodules were ellipsoid shaped, with equal size of the two minor ellipsoid axes. The volume of the nodules corresponded to that of a sphere with a diameter of 4.0, 8.0 or 12.0 mm, resulting in three different nodule size groups, each consisting of 27 nodules. In order to achieve a variety of nodule shapes, ranging from a sphere-like to a more elongated ellipsoid shape, a variation of the ratio of the lengths of the major axis and minor axes was applied. Within each nodule size group, the ratio of the ellipsoid axis lengths was uniformly distributed and ranged from 1.1 to 1.5. For the three size groups, the ranges of the length of the major axis were 4.3–5.2, 8.5–10.5 and 12.8–15.7 mm. In Figure 1, examples of the different nodule shapes are presented. All nodules were assigned homogenous density. In a previous study, Svalkvist *et al.*^(28)^ measured the CT number for a set of real nodules and determined a corresponding attenuation coefficient. The nodules in the present study were assigned an attenuation coefficient of 0.16 cm^−1^, which corresponded to the typical nodule density in the study by Svalkvist *et al*.^(28)^.

### Insertion of nodules into images

A method previously described by Svalkvist *et al.*^(28, 29)^ for simulating the presence of nodules in chest tomosynthesis images was used. With the method, artificial nodules are projected into the raw data of tomosynthesis projection radiographs. The artificial nodules are virtually placed at a desired location within a patient. By extracting information from the digital imaging and communications in medicine (DICOM) header of the projection radiographs regarding focal spot position for each acquisition, and using additional knowledge of the geometry of the tomosynthesis system, the resulting position of the nodule in each of the radiographs is determined. For each projection radiograph, the paths of the X-rays are traced from the focal spot to the detector in order to determine the amount by which they are attenuated by the nodule. In order to take into account the existing anatomy at the desired location of the nodule, the attenuation coefficient of lung tissue is subtracted from the nodule attenuation coefficient. By CT number measurements, and assuming a mean photon energy of 70 kV, Svalkvist *et al.*^(28)^ have previously determined the attenuation coefficient of lung tissue to 0.03 cm^−1^.

The modulation transfer function of the VolumeRAD system is applied to the sampled signal from the simulated nodule in order to take the signal blur in the detector into account in the projection of the nodules. Moreover, the method includes applying a randomised shift in nodule position between the insertions into each of the projection images in order to take blurring due to patient motion into account. The direction of the applied shift in nodule position is randomised according to a uniform distribution over all directions. The magnitude of the shift is randomised according to a normal distribution with a mean of zero and a given standard deviation, referred to as SD_motion_. In order to correct for contrast loss due to scattered radiation, nodule signal strength is adjusted. As a basis for this correction, Monte Carlo simulations of how the scattered radiation varies over different areas of the lung and for the different angles of the projection radiographs with the tomosynthesis system are used^(30)^. Finally, the pixel values of the original projection image are adjusted according to what would be the case if the nodule had actually been present in the patient.

### Simulation of dose reduction

In order to investigate the effect of radiation dose without repeatedly exposing the patients to radiation, a method previously described by Svalkvist and Båth^(31)^ of simulating a dose reduction for a chest tomosynthesis examination was used. The method is an adapted version of an earlier method described for use in radiography at dose levels where the variation in detective quantum efficiency (DQE) is assumed to be small^(32)^. In the case of chest tomosynthesis, the low dose used for the acquisition of the projection radiographs is at a level where the DQE shows a dependency on the dose^(31)^. This is accounted for in the adapted method used in the present study.

The method is based on creating noise images that, when added to the original raw data projection radiographs, will result in reconstructed section images with the same noise power spectrum (NPS) as images actually acquired at a lower dose^(31)^. The method requires that sets of flat-field tomosynthesis projection radiographs are acquired at different dose levels and that the relationship between pixel value and variance as well as the NPS are determined for the flat-field images. The pixel values in the original tomosynthesis radiograph for which the dose reduction is to be applied is scaled according to the desired lower dose level. From the sets of acquired flat-field images, the two images that closest match the mean pixel value of the original radiograph and the scaled version of the original radiograph, and which are matched with the original radiograph according to the angle of projection, are then identified. A noise image is created in the spatial domain by assigning normally distributed, floating point, pseudorandom numbers with a mean of zero and standard deviation of one to the pixels. The noise image is Fourier transformed, and a filter consisting of frequency components determined from the NPS of the two selected flat-field images is applied. Differences in DQE between the dose level of the original radiograph and the dose level of the flat-field image closest matching the original radiograph in dose will result in a loss of proportionality between pixel values and variance between the two dose levels. This also applies to the dose levels of the scaled version of the original radiograph and the flat-field image closest matching the scaled version of the original radiograph in dose. This is corrected for when creating the applied frequency filter by using the previously determined relationship between pixel value and variance. After the filtering of the noise image, it is inversely Fourier transformed. A correction for differences in DQE between the dose levels that exist within the original radiograph is then applied to the pixel values in the noise image. Finally, the noise image is added to the scaled original radiograph. After all projection radiographs have been dose reduced, these are used to create section images with the VolumeRAD reconstruction software.

### Creation of study material

In the study by Svalkvist *et al.*^(28)^ in which the above-described method of simulating the presence of nodules in chest tomosynthesis images was evaluated, nodules with realistic appearances were placed in the lung of a number of patients at locations resembling those of real nodules found in patients. For the present study, 27 of the positions used by Svalkvist *et al*. were selected for the created nodules in the present study. Positions in the lung close to or behind high-density structures such as the heart or the diaphragm were not included. Hence, the selected nodule positions corresponded to locations in high dose areas of the acquired projection radiographs. Nodules from the three different size groups, which corresponded to each other in terms of shape, were placed at identical positions. Any morphological changes that would have occurred in the surrounding anatomy, had the nodule actually been present in the patient, are not accounted for by the method. Therefore, a subjective evaluation of the resulting reconstructed images was made in order to ensure that the nodules were not positioned too close to the structures that would have been affected by the presence of a nodule.

Svalkvist *et al.*^(28)^ determined the value of SD_motion_ for each nodule by visually matching the blurring of the nodule with the blurring of the surrounding anatomical structures in the patient. These values were also used in the present study. For the positions used in the present study, the mean value of SD_motion_ was 0.20 mm and ranged from 0.13 to 0.32 mm.

For all nodules in the present study, the ellipsoid major axis was aligned with the plane of the final reconstructed tomosynthesis section images. All nodules were also centred in the depth direction in one of the section images. In order to introduce a variation in the direction of the major axis in the image plane, the nodules were rotated by a certain amount. The amount of rotation was chosen randomly for each nodule position so that the direction of the major axis was equal for nodules in the three different size groups at identical positions.

The effective dose of the tomosynthesis examination, excluding the scout image, for the average patient included in the present study was estimated to 0.12 mSv, using a previously described method by Båth *et al.*^(33)^ for estimating the dose-area product of a chest tomosynthesis examination and the proposed conversion factor of 0.26 mSvGy^−1^ cm^−2(13)^. The raw data projection images containing the artificial nodules underwent simulated dose reduction using the method^(31)^ described shortly above. For each tomosynthesis image set, two dose reduction simulations were performed, resulting in images corresponding to 50 and 32 % of the original dose level. For the total examination, the simulated dose levels consequently corresponded to effective doses to a standard-sized patient of, respectively, 0.06 and 0.04 mSv, of which the lowest level corresponds approximately to a standard lateral chest radiograph. The raw data images were then used to reconstruct coronal section images at 5-mm intervals using filtered back-projection. The material consisted of 81 tomosynthesis section image series at three different dose levels, including the original dose level, resulting in a total of 243 image series (3 nodule size categories × 3 dose levels × 27 nodules).

**Figure 2.**
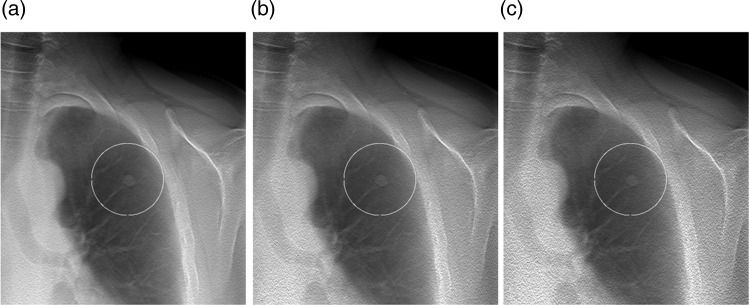
Reconstructed chest tomosynthesis section images included in the study, containing a simulated nodule with the longest diameter of 8.9 mm, at the original dose level (**a**) as well as at a simulated dose level of 50 % (**b**) and 32 % (**c**) of the original dose level.

The study did not include any detection task, and therefore the location of each nodule was marked with a circular region of interest (ROI). All nodules were centred in the ROI, which was present in all section images and was equal in size for all nodules. In Figure 2, an example of a nodule at the three different radiation dose levels included in the study is shown.

### Measurement study

Four radiologists, of which three had ∼20 y of experience in thoracic radiology and 6−7 y of experience in chest tomosynthesis and one had 1 y of experience in both thoracic radiology and chest tomosynthesis, participated in the study. Their task was to determine the longest diameter of all the simulated nodules at all three dose levels. The images were displayed using ViewDEX^(34–36)^, a software designed for use in observer studies with medical images and which includes a tool for performing manual size measurements in the images. For adjustment of the image size during the viewing, a bicubic pixel value interpolation algorithm was used. When assigning values to a pixel in the resampled image, the algorithm uses a cubic spline function of the pixel values in the nearest 4 × 4 pixel grid in the source image^(37)^. The interpolation is performed first along each row of the source image, and after that along each column.

The 81 nodules at the three different dose levels were presented in random order to the observers. In order to investigate intraobserver variability, the nodules were presented in a second round in a new random order. The effect of memory bias within one measurement round or between the first and second measurement rounds was considered small. No instruction to wait a certain time before continuing with the second round was given. The observers were not informed of the size distribution of the nodules but were aware that the nodules were artificial and ellipsoid shaped.

A subset of 10 consecutive section coronal images, in which the nodule was in focus in one of the central images, were presented to the observers. The observers were free to use the magnification tool and adjust the window width and level. For each case and observer, information about in which of the 10 consecutive section images the nodule measurement was performed was logged. The observers were given the possibility of stating a nodule as non-measurable if they deemed the nodule not visible or inadequately reproduced for measurement.

### Analysis of measurement data

The mean of the differences between measurements made in the first measurement round and the actual lengths of the longest nodule diameter was used as a measure of accuracy of nodule size assessment. The accuracy was determined separately for each observer, nodule size group and dose level. Three different sources of measurement variation were used as measures of the precision of the nodule size assessment^(19)^: (1) The intraobserver variability, expressing the inherent variation in repeated measurements on the same nodule by one observer, was determined as the standard deviation of the two measurements on each nodule by each observer, averaged over all nodules in each size group and dose level. (2) The interobserver variability, expressing the variation in measurements on the same nodule by multiple observers, was determined as the standard deviation of the measurements by all observers on each nodule, averaged over all nodules in each size group and dose level. (3) The internodule variability, expressing the measurement variation due to the anatomy surrounding the nodules and the different attributes of the nodules, was determined for all observers and dose levels as the standard deviation of the differences between measurements made in the first measurement round and the actual lengths of the longest nodule diameter for all nodules in each size group. The interobserver and internodule variability will include the intraobserver variability; hence, the three measures of nodule measurement precision are not independent. The standard deviations expressing the intraobserver and interobserver variabilities were based on small sample sizes (sample sizes 2 and 4). Therefore, a correction factor was applied in the determination of these variabilities^(38)^. The uncertainty of each measure of measurement accuracy and precision was expressed with 95 % confidence intervals. For each observer, dose level and size group, the number of nodules judged as non-measurable was determined. If, during analysis of the measurement data of the observers in conjunction with visual inspection of the images, it was judged that a measurement had been made on a structure other than a nodule, this measurement was excluded from further analysis of accuracy and precision.

Repeated measures analysis of variance (ANOVA) was used to test the overall effect of dose level on the mean of the absolute values of the differences between measurement results and the actual lengths of the longest diameter, as well as on intraobserver and interobserver variabilities. The tests were made separately for each nodule size group and observer. For each observer and nodule size group, only nodules for which there were measurements at all three dose levels were included in the ANOVA test. If the ANOVA test resulted in a significant overall effect, *post hoc* pairwise comparisons were made using a Bonferroni correction for multiple comparisons^(39)^. As a measure of association between dose level and internodule variability, the Kendall rank correlation coefficient, referred to as Kendall's tau, was used. A significance level of 5 % was chosen.

## RESULTS

The number of nodules judged as non-measurable by the observers for each dose level and nodule size group is presented in Table 1. Of the smallest nodules, the number judged as non-measurable ranged from 6 to 14 for all dose levels. For all observers, the number of the smallest nodules judged as non-measurable was higher for the two lower dose levels compared with the original dose level. For the nodules corresponding in volume to a sphere of 8.0 mm, all nodules at the original dose level were found measurable, while two observers found in total four nodules non-measurable at the two lower dose levels. All observers found all of the largest nodules measurable at all dose levels.

**Table 1.** Number of nodules, in each nodule size group and dose level, judged as non-measurable by the observers.

Observer	Dose level (%)	Non-measurable in the smallest size group (*n*)	Non-measurable in intermediate size group (*n*)	Non-measurable in the largest size group (*n*)
1	100	6	—	—
	50	12	—	—
	32	10	—	—
2	100	11	—	—
	50	12	1	—
	32	12	2	—
3	100	9	—	—
	50	14	—	—
	32	12	1	—
4	100	7	—	—
	50	13	—	—
	32	12	—	—

The total number of nodules in each size group was 27.

**Figure 3.**
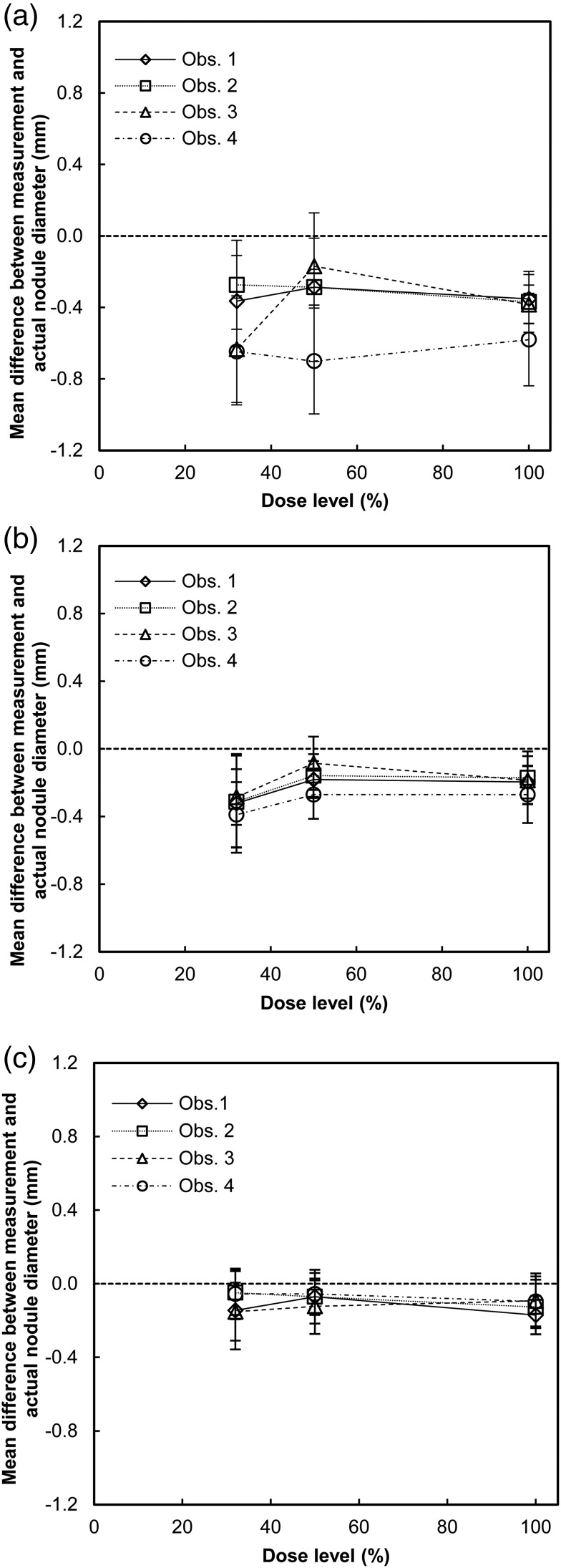
Measurement accuracy for the four observers and three dose levels for nodules corresponding in volume to a sphere with a diameter of (**a**) 4 mm, (**b**) 8 mm and (**c**) 12 mm. Uncertainty bars represent the 95 % confidence intervals.

The accuracy of nodule size assessment, defined as the mean of the differences between measurements made in the first measurement round and the actual lengths of the longest nodule diameter, for all observers, nodule size groups, and dose levels, are presented in Figure 3. For all observers, nodule sizes and dose levels, there was a tendency for a small underestimation of the nodule size. The accuracy ranged from approximately −0.7 to −0.2, −0.4 to −0.1 and −0.2 to −0.1 mm for the smallest, intermediate-sized and the largest nodules, respectively. The repeated measures ANOVA determined that there was a significant effect of dose level on the mean of the absolute values of the differences between measurement results and the actual lengths of the longest diameter for Observer 3 for the smallest nodules (*F*(2, 16) = 11.676, *p* = 0.001), as well as for Observer 1 (*F*(2, 52) = 8.426, *p* = 0.001) and Observer 2 (*F*(2, 46) = 3.851, *p* = 0.028) for the intermediate-sized nodules. *Post hoc* tests showed that for Observer 3 the mean at the lowest dose level (0.67±0.22 mm) was significantly larger than at the 50 % dose level (0.29±0.11 mm) and at the 100 % dose level (0.28±0.14 mm) (*p* = 0.008 and *p* = 0.019, respectively) for the smallest nodules. For Observer 1, the *post hoc* test showed that the mean at the lowest dose level (0.60±0.21 mm) was significantly larger than at the 50 % dose level (0.27±0.07 mm) and at the 100 % dose level (0.24±0.08 mm) (*p* = 0.036 and *p* = 0.009, respectively) for the intermediate-sized nodules. For Observer 2, the *post hoc* test did not show any significant difference between any of the pairs of dose levels. For the largest nodules, no significant effect of the dose level on the difference between the mean of the absolute values of the differences between measurement results and the actual lengths of the longest diameter was found for any of the observers.

**Figure 4.**
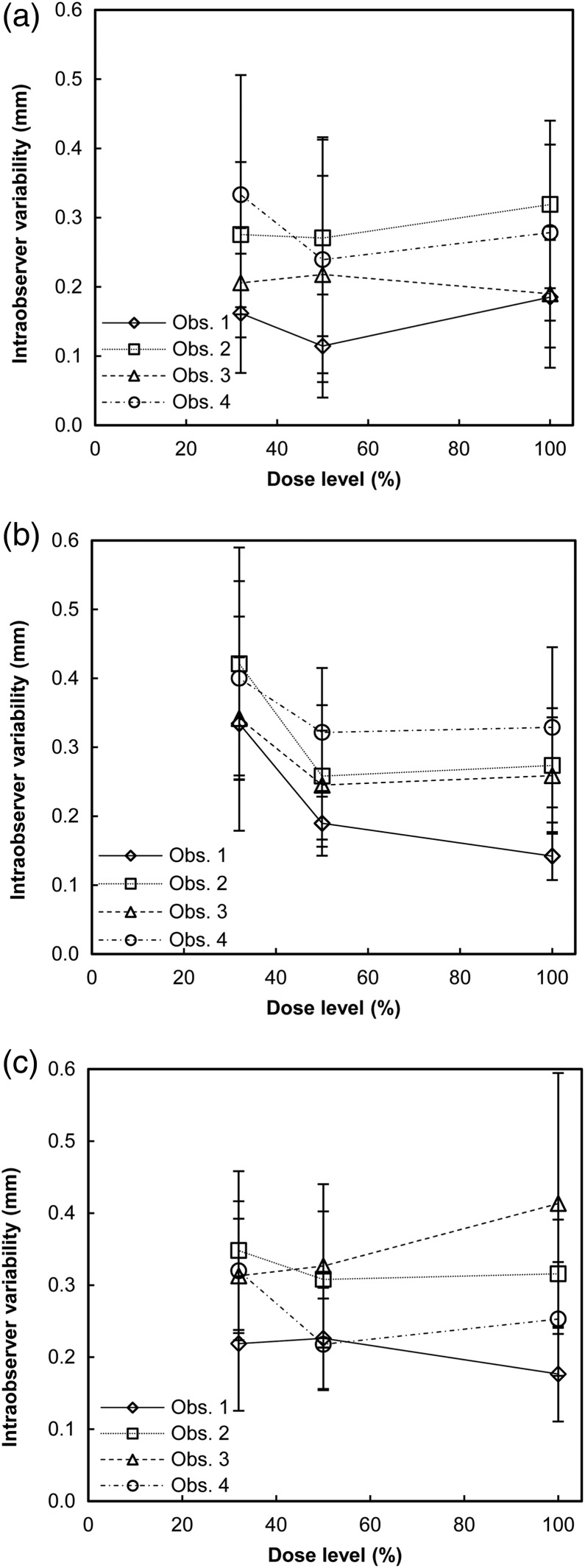
Intraobserver variability, defined as the mean of the standard deviations of the pairs of measurements on the same nodule in each nodule size group, for the four observers and three dose levels for nodules corresponding in volume to a sphere with a diameter of (**a**) 4 mm, (**b**) 8 mm and (**c**) 12 mm. Uncertainty bars represent the 95 % confidence intervals.

**Figure 5.**
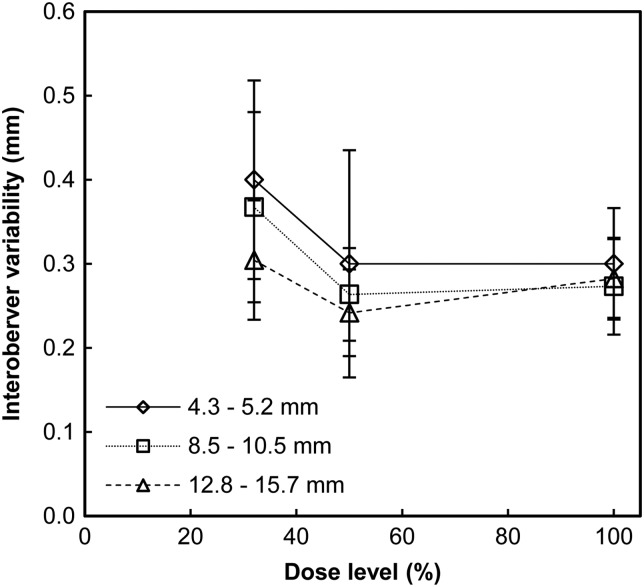
Interobserver variability, defined as the mean of the standard deviations of the measurements by all observers on the nodules in each size group, for the three dose levels. Uncertainty bars represent the 95 % confidence interval.

**Figure 6.**
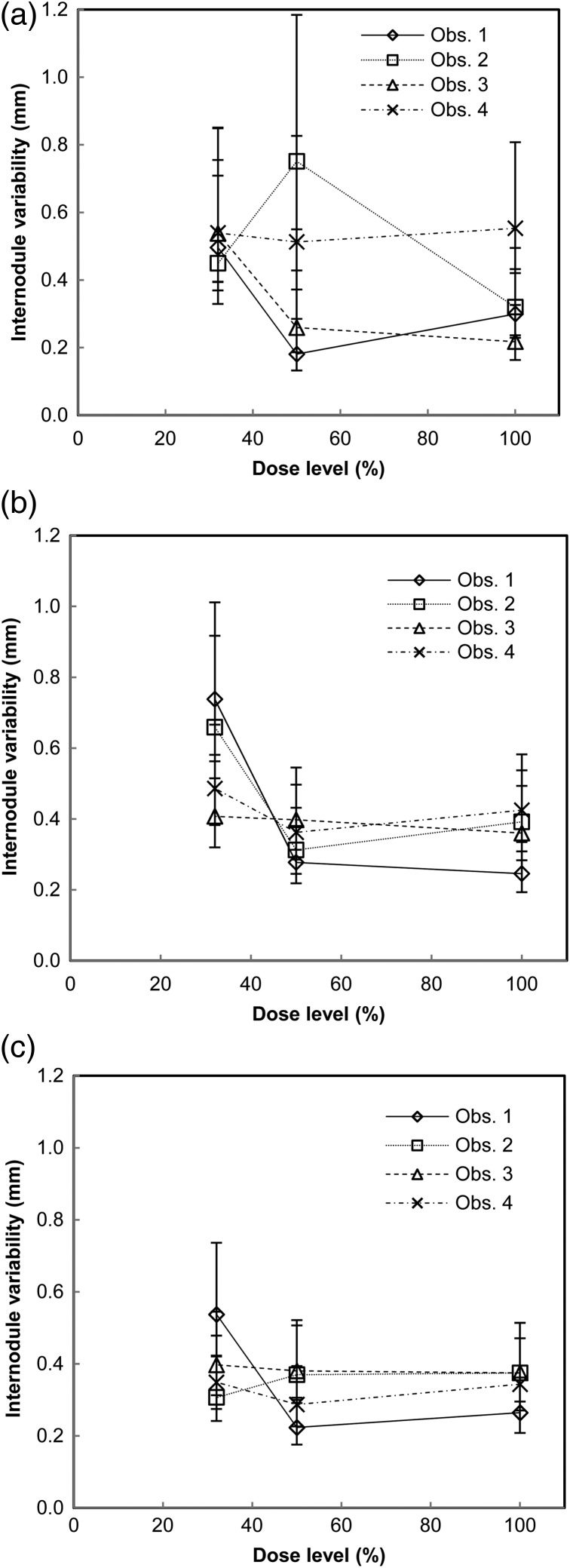
Internodule variability, defined as the standard deviation of all measurements on nodules in each nodule size group, for the four observers and three dose levels for nodules corresponding in volume to a sphere with a diameter of (**a**) 4 mm, (**b**) 8 mm and (**c**) 12 mm. Uncertainty bars represent the 95 % confidence intervals.

The intraobserver, interobserver and internodule variabilities are presented in Figures 4–6, respectively. The intraobserver variability was of similar magnitude for the three different nodule size groups. For all dose levels and observers, the intraobserver variability ranged from ∼0.1 to 0.3, 0.1 to 0.4 and 0.2 to 0.4 mm for the smallest, intermediate-sized and the largest nodules, respectively. For the intraobserver variability, the ANOVA test determined significant effect of the dose level for Observer 1 for the intermediate-sized nodules (*F*(2, 48) = 5.26, *p* = 0.009). The *post hoc* tests determined a significantly larger intraobserver variability at the lowest dose level (0.33±0.16 mm) than at the 100 % dose level (0.14±0.04 mm) (*p* = 0.036). The interobserver variability ranged from ∼0.2 to 0.4 mm for all nodule sizes and dose levels. For all nodule sizes, the interobserver variability was highest at the lowest dose level. However, no significant effect of dose levels on the interobserver variability for any of the nodule size groups was determined with the ANOVA test. The similar magnitudes of the intraobserver and interobserver variabilities indicated relatively small differences between observers. For all dose levels and observers, the internodule variability ranged from ∼0.2 to 0.8, 0.2 to 0.7 and 0.2 to 0.5 mm for the smallest, intermediate-sized and the largest nodules, respectively. Kendall's tau was determined to −1.0 (*p* < 0.001) for Observer 1 for the smallest nodules, for Observers 1 and 3 for the intermediate-sized nodules, and for Observer 3 for the largest nodules, indicating a negative association between dose level and internodule variability for these observers and nodule sizes. For Observer 2, the Kendall's tau was determined to 1.0 (*p* < 0.001) for the largest nodules, indicating a positive association between dose level and internodule variability. The somewhat larger internodule variability, when compared with the intraobserver variability, indicates that the nodule features and the position of the nodule are a source of measurement variation.

## DISCUSSION

Follow-up of incidentally detected pulmonary nodules, usually done by repeated CT scans, has been suggested as a clinical task for which chest tomosynthesis might be suitable^(3)^. This suggestion has been supported by studies indicating high nodule size measurement accuracy in chest tomosynthesis images as well as good agreement between measurements made on chest tomosynthesis and CT images^(17–20)^. Studies have also shown that a substantial reduction in current, commonly used, dose levels for chest tomosynthesis might be possible without significantly reducing the detection of pulmonary nodules^(25, 26)^. The results of the present study suggest that a radiation dose level resulting in an effective dose of 0.06 mSv for a standard patient could be used for chest tomosynthesis examinations without significantly affecting the accuracy or precision of measurements of the longest diameter of pulmonary nodules. It should be emphasised that the present study was restricted to nodules located in high-dose regions of chest tomosynthesis section images. As a previous study by Söderman *et al.*^(19)^ has indicated that nodules located in regions corresponding to low-dose areas in the acquired projection radiographs, such as behind the heart or the diaphragm, suffer from low visibility and decreased size measurement accuracy, it can be argued that chest tomosynthesis is less suitable for follow-up of nodules located in these regions.

In order to investigate possible systematic measurement errors with chest tomosynthesis in the present study, simulated ellipsoid-shaped nodules with known dimensions were created and inserted into clinical chest tomosynthesis images so that the ellipsoid major axis was parallel to the plane of the reconstructed tomosynthesis section images. Moreover, all nodules were centred in the depth direction of one of the section images. An ideal condition for assessing the actual size of the longest diameter of the nodules was thus created, and the uncertainty in the clinical situation of matching the longest diameter of the nodule to the plane of image reconstruction was not included in the present study. In the clinical setting, the probability of including the longest diameter in the reconstructed section images can be increased by reconstructing the section images at smaller intervals than was done in the present study.

For all nodule size groups and dose levels, the longest diameter was on average underestimated by all observers. The largest mean for an observer and size group was −0.7 mm. Slight underestimation of nodule diameters has been reported previously in studies investigating manual diameter measurements in chest tomosynthesis at standard dose levels using artificial nodules with known sizes^(17, 19, 20)^.

Regarding the precision of the nodule diameter measurements, the magnitude of the variability measures was of similar magnitude to what has been presented by Söderman *et al.*^(19)^ in a previous study performed at the standard dose level for chest tomosynthesis. Except for one observer for the intermediate-sized nodules, no significant effect of the dose level on the intraobserver variability could be found in the present study. The largest interobserver variability was seen for the lowest radiation dose level for all nodule size groups. However, no significant effect of the dose level on the interobserver variability could be seen. The overall levels of interobserver variabilities were small, with the highest interobserver variability being 0.4 mm, and can be argued to be of little clinical relevance.

The relatively large number of nodules judged as not adequately visible for making reliable diameter measurements on in the smallest size group at all dose levels introduces some concerns of the use of chest tomosynthesis for follow-up for nodules with a diameter of 5 mm or smaller. Previous studies have shown that the visibility of these smaller nodules can be limited^(19, 25)^. In a previous study by Asplund *et al.*^(25)^ investigating nodule detection in chest tomosynthesis, it was found that of nodules measuring 4 mm or less in chest CT images with a slice thickness of 1.25 or 0.6 mm, only 52 % were visible in retrospect in chest tomosynthesis images at a dose level corresponding to the original dose level in the present study. The risk of missing new smaller nodules with follow-up using chest tomosynthesis has previously been expressed by Dobbins and MacAdams^(3)^. For the intermediate-sized nodules in the present study, the overall visibility was high and the effect of dose on visibility was very small. All of the largest nodules were visible at all dose levels. For the smaller nodules that were measurable in the present study, the measurement precision was of similar magnitude as that of the larger nodules. This indicates that for a smaller nodule detected in a chest tomosynthesis examination and judged as measurable, follow-up with tomosynthesis might be achievable. However, it has been suggested that not every nodule with a diameter of 5 mm or less at the time of detection requires follow-up due to that the probability of malignancy has been shown to be <1 % for such a nodule^(16, 22)^.

A method for simulating dose reduction of tomosynthesis examinations, previously described by Svalkvist and Båth^(31)^, was used in the present study in order to investigate any dependency of the measurement accuracy and precision on the resulting effective dose to the patient. With the tomosynthesis system used in the present study, the DQE of the detector is reduced when the dose is lowered, primarily due to a relative increase of the electronic noise of the system as the dose is reduced^(31)^. As discussed by Asplund *et al.*^(25)^, this will introduce a higher level of image noise than might be expected. Additionally, Asplund *et al.* pointed out that as the DQE will decrease more quickly for higher spatial frequencies at lower doses, lower dose level images will include more fine-structured noise. This could be an additional reason for the somewhat lower visibility of the smaller nodules at the two lower dose levels compared to the original dose level.

The dose levels evaluated in the present study, in addition to the original dose level, were 50 and 32 % of the original dose. The 32 % level corresponds to that of a conventional lateral view radiograph acquired with the system used in the present study^(13)^. At this level, the accuracy and precision were at clinically accepted levels when compared with the original dose level in the present study. If a reduction of the effective dose of a chest tomosynthesis examination, to the same level as a lateral view radiograph, were implemented in the clinic, a tomosynthesis examination including the scout would correspond in effective dose to that of a conventional chest radiography examination, including a PA and lateral view radiograph.

It has been found that the computer code used for implementation of the method for inserting artificial nodules in images includes a feature of a rounding function that has not been accounted for^(40)^. Under certain circumstances, this results in an erroneous reproduction of the size of nodules^(40)^. The effect is particularly apparent for nodules located in low-dose areas of the tomosynthesis projection radiographs. However, for the nodule positions included in the present study, the effect of the error in the code was by visual inspection, performed by an experienced thoracic radiologist, deemed negligible in terms of apparent size and visibility of the nodule, when nodules inserted with the erroneous code and a corrected version of the code^(40)^ were compared. The method for simulating the presence of nodules in chest tomosynthesis was also used in a similar study including nodule positions in both high- and low-dose regions of the images^(19)^. In that study, however, the corrected version of the code was used, resulting in no bias by the error.

The use of ellipsoid-shaped artificial nodules with smooth surfaces was a limitation of the present study. The variability of measurements made on real pulmonary nodules found in patients will be affected by the varying shapes and irregular surfaces of real nodules, which were not accounted for here. The nodules were given smooth surfaces as a way of knowing exactly the longest diameter of the nodules, which allowed for an analysis of measurement accuracy. Using ellipsoid-shaped nodules enabled the possibility of controlling in which direction in the image plane the longest diameter was oriented.

The primary objective of pulmonary nodule follow-up is to detect any growth of the nodule over time that could indicate malignancy. In the present study, no investigation of the possibility of detecting size changes with chest tomosynthesis was performed. As a nodule can grow asymmetrically, the sensitivity of chest tomosynthesis in detecting size change may be limited by the fact that nodule measurements are restricted to only one plane, as opposed to CT. CT also allows for automated volumetric measurements of nodules, which at present is not an option for chest tomosynthesis. However, methods for achieving a more isotropic resolution in tomosynthesis, by adding a second tomosynthesis acquisition from another view, have recently been presented^(41, 42)^. Nevertheless, before chest tomosynthesis can be used for pulmonary nodule follow-up, its ability to detect actual size changes should be analysed.

## CONCLUSION

For pulmonary nodules at positions in the lung corresponding to locations in high-dose areas of the projection radiographs, using a radiation dose level resulting in an effective dose of 0.06 mSv to a standard-sized patient may be possible in chest tomosynthesis without affecting the accuracy and precision of nodule diameter measurements to any large extent. However, an increasing number of non-measurable small nodules (≤5 mm) with decreasing radiation dose may raise some concerns regarding an applied general dose reduction in the clinical praxis for chest tomosynthesis examinations.

## FUNDING

This work was supported by grants from the Swedish Research Council (2011/488, 2013/3477), the Swedish Radiation Safety Authority (2014/2641), the Swedish Federal Government under the LUA/ALF agreement (ALFGBG-428961) and the Health and Medical Care Committee of the Region Västra Götaland (VGFOUREG-483951).
